# The Effect of Flavonoid Aglycones on the CYP1A2, CYP2A6, CYP2C8 and CYP2D6 Enzymes Activity

**DOI:** 10.3390/molecules24173174

**Published:** 2019-09-01

**Authors:** Mirza Bojić, Martin Kondža, Hrvoje Rimac, Goran Benković, Željan Maleš

**Affiliations:** 1 Department of Pharmaceutical Chemistry, Faculty of Pharmacy and Biochemistry, University of Zagreb, A. Kovačića 1, 10000 Zagreb, Croatia; 2 Matice hrvatske, Faculty of Pharmacy, University of Mostar, 88000 Mostar, Bosnia and Herzegovina; 3 Agency for Medicinal Products and Medical Devices, Ksaverska cesta 4, 10000 Zagreb, Croatia; 4 Department of Pharmaceutical Botany, Faculty of Pharmacy and Biochemistry, University of Zagreb, Schrottova 39, 10000 Zagreb, Croatia

**Keywords:** flavonoids, CYP1A2, CYP2A6, CYP2C8, CYP2D6, inhibition

## Abstract

Cytochromes P450 are major metabolic enzymes involved in the biotransformation of xenobiotics. The majority of xenobiotics are metabolized in the liver, in which the highest levels of cytochromes P450 are expressed. Flavonoids are natural compounds to which humans are exposed through everyday diet. In the previous study, selected flavonoid aglycones showed inhibition of CYP3A4 enzyme. Thus, the objective of this study was to determine if these flavonoids inhibit metabolic activity of CYP1A2, CYP2A6, CYP2C8, and CYP2D6 enzymes. For this purpose, the *O*-deethylation reaction of phenacetin was used for monitoring CYP1A2 enzyme activity, coumarin 7-hydroxylation for CYP2A6 enzyme activity, 6-α-hydroxylation of paclitaxel for CYP2C8 enzyme activity, and dextromethorphan *O*-demethylation for CYP2D6 enzyme activity. The generated metabolites were monitored by high-performance liquid chromatography coupled with diode array detection. Hesperetin, pinocembrin, chrysin, isorhamnetin, and morin inhibited CYP1A2 activity; apigenin, tangeretin, galangin, and isorhamnetin inhibited CYP2A6 activity; and chrysin, chrysin-dimethylether, and galangin inhibited CYP2C8. None of the analyzed flavonoids showed inhibition of CYP2D6. The flavonoids in this study were mainly reversible inhibitors of CYP1A2 and CYP2A6, while the inhibition of CYP2C8 was of mixed type (reversible and irreversible). The most prominent reversible inhibitor of CYP1A2 was chrysin, and this was confirmed by the docking study.

## 1. Introduction

Cytochrome P450 (CYP) enzymes are the most significant enzymes for the metabolism of substances foreign to the human body, including drugs [1]. These enzymes are hemoproteins containing heme, which is bound to the apoprotein part. The iron ion is linked to the heme by four coordinative covalent bonds, while the fifth coordinative covalent bond links iron to the cysteine residue of the apoprotein. Functionally cytochromes P450 are monooxygenases that incorporate one atom of oxygen from molecular oxygen into the substrate [1].

The reactions catalyzed by cytochromes P450 are not limited to one type of reaction or one substrate. Instead, each cytochrome P450 enzyme has numerous substrates and catalyzes chemically diverse reactions. Beside oxidation reactions (e.g., hydroxylations, dealkylations), cytochromes catalyze reduction, desaturation, ester cleavage, ring expansion, ring formation, aldehyde scission, dehydration, ipso attack, one-electron oxidation, coupling reactions, rearrangement of fatty acid, and prostaglandin hydroperoxides [2].

The major site of cytochromes P450 activity within the human organism is the liver. The liver cytochromes P450 involved in the metabolism of most drugs are: CYP3A4/5 (30%), CYP2D6 (20%), CYP2C9 (13%), CYP1A2 (9%), and others [3]. As nearly 94% of oxidation reactions of medicinal drugs are catalyzed by cytochromes P450, the involvement of these enzymes in the pharmacokinetic of the drugs is significant.

Drugs and other xenobiotics can be substrates of cytochromes P450, but they can also act as inhibitors. Two substrates competing for the same active site of the cytochrome P450 enzyme present the most common observed interaction, e.g., ketoconazole is a competitive CYP3A4 inhibitor that can reduce terfenadine metabolism, which is almost exclusively metabolized by CYP3A4 [4]. The aforementioned interaction is reversible, i.e., discontinuation of the ketoconazole application normalizes terfenadine metabolism and pharmacokinetic.

Inhibitions that can have more significant consequences are irreversible. These are usually caused by inactivation of cytochromes P450 by a reactive intermediar that covalently binds to the apoprotein or the heme part of cytochrome P450. A good example is the antihypertensive drug mibefradil, which irreversibly inhibits CYP3A4. Mibefradil is converted to a reactive intermediar in the catalytic cycle of CYP3A4. The generated intermediar covalently binds to the heme part of CYP3A4, causing the inactivation of the enzyme [5]. The enzyme activity is lost and can only be regained by the expression of an additional protein (enzyme), which takes days to weeks [6].

Except drugs, other xenobiotics like flavonoids can interact with cytochrome P450. Flavonoids are natural polyphenols abundantly present in higher plants, including fruits, vegetables, and plant-derived products, such as wine and propolis [7,8,9]. Flavonoids are regarded as vitamins that are important in the regulation of oxidative stress and act as antioxidants. Consequently, flavonoids have many beneficial health effects such as antiallergic, anti-inflammatory, antioxidative, antimicrobial, antitumorigenic, and antimutagenic effects, thus preventing cancer, heart disease, bone loss, and a number of other diseases [8,10,11,12,13].

Flavonoids, in nature, mainly come bound to a sugar moiety as glycosides. Glycosides are susceptible to hydrolysis, and aglycones are liberated and absorbed in the gut [6]. In the liver, aglycones are metabolized by the cytochromes P450. The major metabolic reactions to which flavonoids are susceptible are aromatic hydroxylations and *O*-dealkylations, which are catalyzed by cytochromes P450 [14,15]. The major cytochromes P450 involved in the metabolism of flavonoid aglycones are CYP1A2, CYP3A4, and CYP2D6 [14,15]. Flavonoids can also act as inhibitors of metabolic enzymes, causing clinically significant interactions [16,17,18,19,20,21].

**Figure 1:**
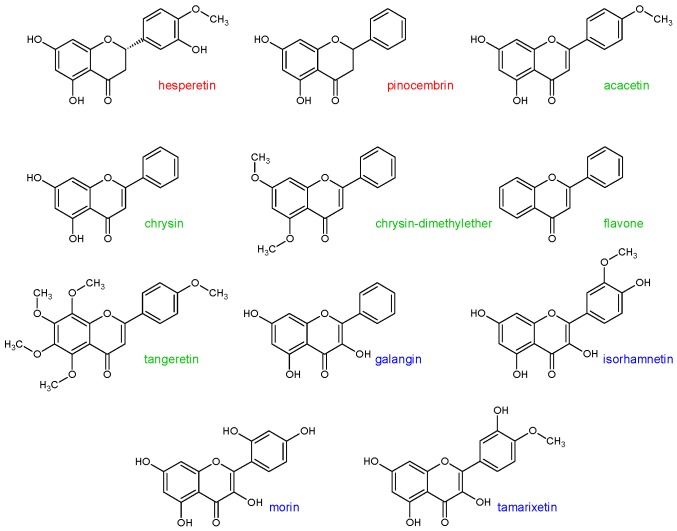
Structural characteristics of flavonoids used in this study. Flavanones are marked in red, flavones in green, and flavanols in blue.

In the previous study [22], it was shown that some flavonoid aglycones can cause the inhibition of CYP3A4 metabolic activity. These flavonoids were: Hesperetin, pinocembrin, acacetin, chrysin, chrysin-dimethylether, flavone, tangeretin, galangin, isorhamnetin, morin, and tamarixetin (statistical significance *p* < 0.1) (Figure 1). Thus, the aim of this study was to assess the inhibitory effect of the aforementioned flavonoids on the CYP1A2, CYP2A6, CYP2C8, and CYP2D6 enzymes activity.

## 2. Results and Discussion

**Figure 2:**
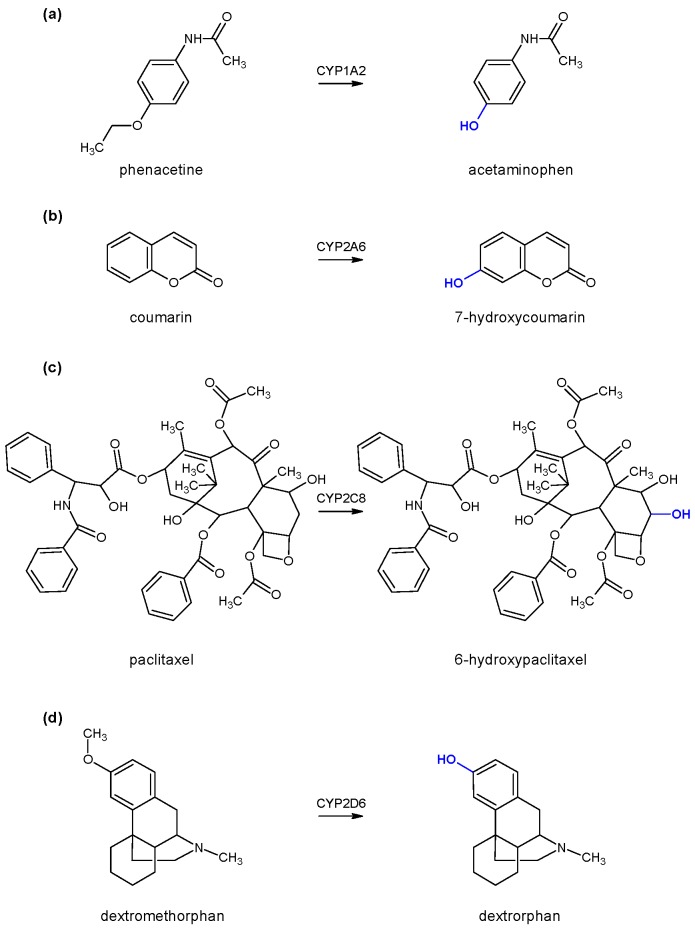
Marker substrates and reactions used for monitoring enzyme activity: (**a**) Phenacetin *O*-deethylation for the CYP1A2 enzyme, (**b**) coumarin 7-hydroxylation for the CYP2A6 enzyme, (**c**) paclitaxel 6-α-hydroxylation for the CYP2C8 enzyme, and (**d**) dextromethorphan *O*-demethylation for the CYP2D6 enzyme. The sites of reaction are marked in blue.

To test the enzyme activity of each individual cytochrome P450, marker substrates/reactions were used, namely phenacetin *O*-deethylation for monitoring CYP1A2 enzyme activity, coumarin 7-hydroxylation for monitoring CYP2A6 enzyme activity, paclitaxel 6-α-hydroxylation for monitoring CYP2C8 enzyme activity, and dextromethorphan *O*-demethylation for monitoring CYP2D6 enzyme activity (Figure 2). All generated products were determined using high-performance liquid chromatography coupled with a diode-array detector (HPLC-DAD) with the appropriate method of analysis for each individual reaction (*vide infra*).

**Figure 3:**
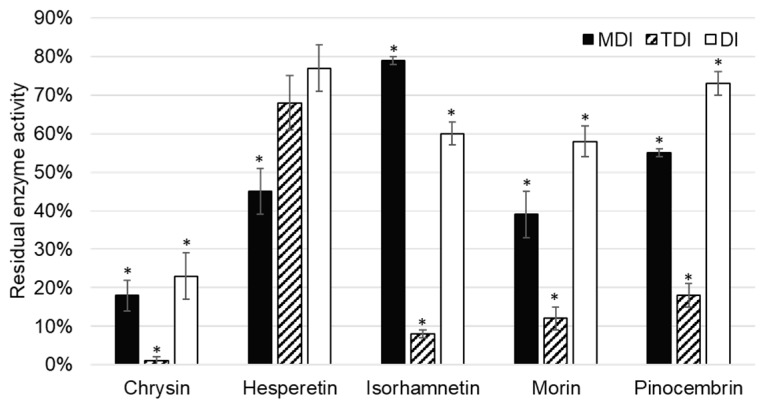
Residual CYP1A2 enzyme activity after incubation with 1 µM flavonoid in the experiments for the determination of metabolism dependent inhibition (MDI), time dependent inhibition (TDI), and direct inhibition (DI). * denotes that observed inhibition is statistically significant (*p* < 0.05) when compared to the control (without addition of flavonoid).

CYP1A2 activity was inhibited by five of the tested flavonoids, namely chrysin, hesperetin, isorhamnetin, morin, and pinocembrin (Figure 3).

Chrysin showed inhibition of CYP1A2 in all inhibition assays: Metabolism (MDI) and time dependent (TDI), as well as in the direct inhibition (DI) assay. As direct inhibition and metabolism inhibition assay are of comparable residual activities (23 ± 6% (*p* = 0.001) and 18 ± 4% (*p* = 0.004), respectively), it can be concluded that inhibition is reversible. This is in accordance with previously published data. Lee et al. [23] tested 21 flavonoids, out of which chrysin was shown to be the strongest inhibitor of CYP1A2. The enzyme activity was tested with *N*^3^-demethylation of caffeine as a marker reaction, and chrysin showed reversible, competitive inhibition. On rat liver microsomes, Siess et al. [24] showed that chrysin inhibited ethoxy- and pentoxy-resorufin dealkylation and characterized the inhibition as mix type reversible. Phenacetin *O*-deethylation and caffeine *N*^3^-demethylation are marker reactions of CYP1A2, ethoxy- and pentoxy-resorufin, while dealkylation was used to characterize the overall contribution of all cytochromes. The inhibition observed in the study performed by Siess et al. [24] can be consequence of the inhibition of other cytochromes P450, rather than CYP1A2. However, Kim et al. [25] confirmed the reversible mix inhibition of CYP1A2 using the ethoxyresorufin *O*-deethylation reaction on a recombinant cytochrome P450 system. Mixed inhibition is in accordance with observed results of the TDI assay that showed complete inhibition of CYP1A2 (Figure 3). In the TDI assay, sufficient time is given by preincubating chrysin with the enzyme before starting the catalytic marker reactions.

Hesperetin showed a less extensive inhibition of CYP1A2 under the same conditions compared to other flavonoid inhibitors of CYP1A2. As residual activity increased in the order of DI, TDI, and MDI assays, it can be concluded that hesperetin showed reversible, as well as irreversible, inhibition of CYP1A2, although the later one was not statistically significant (Figure 3). Doostdar et al. [26] showed that hesperetin is an inhibitor of CYP1B1, while residual CYP1A2 enzyme activity was not statistically different to control in the analyzed range of concentrations, including the one used in the present study (1 µM). This difference could be attributed to different marker reaction used by Doostdar et al. [26], i.e., ethoxyresorufin *O*-dealkylation. The significance of hesperetin CYP1A2 inhibition was relevant for the pharmacokinetic and metabolism of rasagiline mesylate in Wistar rats, explaining the observed drug-hesperetin interaction [27].

Chang et al. [28] showed competitive inhibition of CYP1A2-mediated 7-ethoxyresorufin *O*-dealkylation with an inhibition constant of 0.14 µM. This is in accordance with results of our study, in which isorhamnetin was shown to be a reversible inhibitor of CYP1A2. Interestingly, if isorhamnetin is preincubated with CYP1A2, the residual activity of CYP1A2 decreases to 8 ± 2% (*p* = 0.012).

On rat liver microsomes, Siess et al. [24] showed that morin inhibited ethoxy- and pentoxy-resorufin dealkylation, and characterized the inhibition as mix type reversible similarly to chrysin. However, the inhibition constant for morin was 16-fold greater (5 µM). This is in accordance with the results of our study, as patterns observed in MDI, TDI, and DI assays were the same for morin and chrysin, and chrysin was a more potent inhibitor than morin. Sahu et al. [29] showed that the inhibition of CYP1A2 could influence the febuxostat metabolism (substrate of CYP1A2), causing drug-flavonoid interactions, while Li et al. [30] confirmed interactions of etoposide and morin, although later one was attributed to the other cytochromes P450 as well.

Pinocembrin inhibited CYP1A2 enzyme activity. The observation that pinocembrin acts as a metabolism dependent, time dependent, and direct inhibitor of CYP1A2 has not been previously reported. As in the case of the most of aforementioned flavonoid inhibitors of CYP1A2, time dependent inhibition is the most prominent compared to other types. This indicates that these flavonoids require time to interact with CYP1A2 for the inhibition to be observed, and could be attributed to allosteric, nonspecific binding to the enzyme.

**Figure 4:**
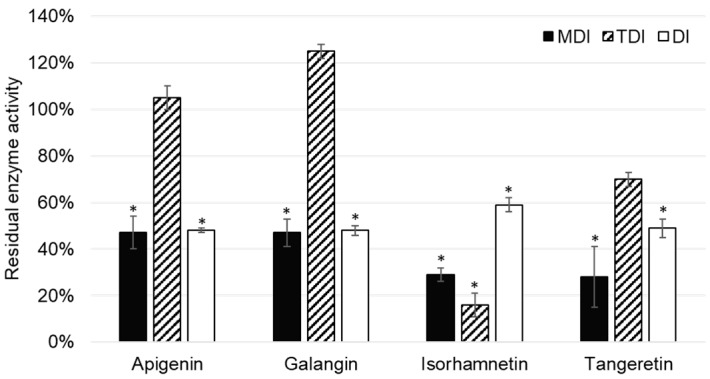
Residual CYP2A6 enzyme activity after incubation with 1 µM flavonoid in the experiments for the determination of metabolism dependent inhibition (MDI), time dependent inhibition (TDI), and direct inhibition (DI). * denotes that observed inhibition is statistically significant (*p* < 0.05) when compared to the control (without addition of flavonoid).

Out of 11 analyzed flavonoids, apigenin, galangin, isorhamnetin, and tangeretin inhibited CYP2A6 enzyme activity (Figure 4). Although TDI was an important type of CYP1A2 enzyme, this was not the case with the CYP2A6 enzyme, as it was shown only to be significant in the case of isorhamnetin.

Apigenin was reported to inhibit CYP2A6 activity in the metabolism dependent inhibition assay, as well as the direct inhibition assay of CYP2A6. Boonruanga et al. [31] reported similar *IC*_50_ values in metabolism dependent and direct inhibition assays of 0.77 ± 0.16 µM and 0.90 ± 0.07 µM, respectively. Likewise, under the similar experimental conditions and 1 µM concentration of apigenin, we observed comparable values of residual CYP2A6 enzyme activity: 47 ± 7% (*p* = 0.037) and 48 ± 1% (*p* = 0.018). This indicates that apigenin is a direct, reversible inhibitor of the CYP1A2 enzyme.

Galangin showed similar values of residual CYP2A6 activity in MDI and DI assays, indicating that it is a reversible, direct inhibitor of CYP1A2. This is in accordance with the results of Tiong et al. [32], who conducted experiments using the recombinant enzyme and the same marker reaction as our study.

Isorhamnetin showed inhibition of CYP2A6 enzyme activity, which was most prominent in the TDI assay: 16 ± 5% (*p* = 0.025). (Figure 4). This indicates that isorhamnetin acts as a reversible, time dependent inhibitor of CYP1A2. This could explain the observed interactions of the *Ginkgo biloba* extract with valproic acid observed by Numa et al. [33].

Although it has been reported that tangeretin is metabolized by cytochromes P450, namely CYP1A2, CYP3A4, and CYP2D6 [14,15], it is interesting to note that tangeretin acts as a direct inhibitor of CYP2A6 enzyme activity.

**Figure 5:**
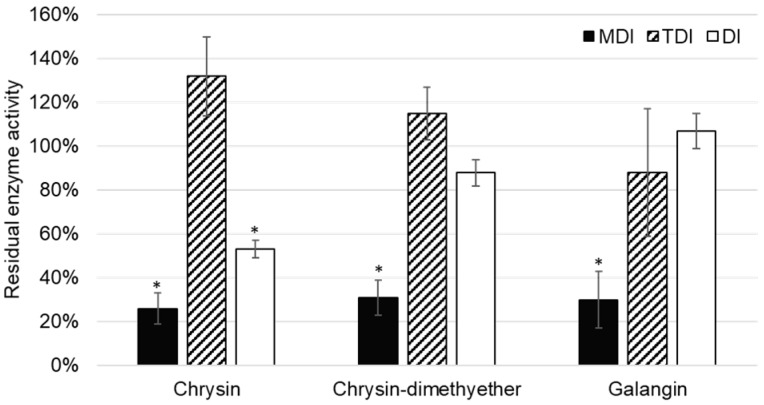
Residual CYP2C8 enzyme activity after incubation with 1 µM flavonoid in the experiments for determination of metabolism dependent inhibition (MDI), time dependent inhibition (TDI), and direct inhibition (DI). * denotes that observed inhibition is statistically significant (*p* < 0.05) when compared to the control (without addition of flavonoid).

Although the inhibition of CYP2C8 was reported for some flavonoids [34,35], this is the first report on the inhibition of CYP2C8 paclitaxel 6-α-hydroxylation by chrysin, chrysin-dimethylether, and galangin. In the TDI assay, the reduction of enzyme activity was not observed, and values of residual CYP2C8 enzyme activity were lower in MDI compared to DI assay (Figure 5). Thus, it can be concluded that chrysin, chrysin-dimethylether, and galangin are mixed reversible and irreversible inhibitors of CYP2C8.

Interestingly, no inhibition of CYP2D6 was observed on the set of analyzed flavonoids, neither reversible nor irreversible. Flavonoids are weak acids, having mainly hydroxyl groups, while typical CYP2D6 substrates contain a nitrogen atom, which can be protonated at physiological pH [36]. This could explain why not even direct competitive inhibition was observed when dextromethorphan was used as the marker substrate of CYP2D6 activity.

In our previous research, a significant number of irreversible flavonoid inhibitors of CYP3A4 was reported [22]. However, flavonoids in this study mainly served as reversible inhibitors of CYP1A2 and CYP2A6, while the inhibition of CYP2C8 was of mixed type (reversible and irreversible).

To assess the reversible binding of flavonoids to cytochromes P450, a docking study of the most potent reversible inhibitor, i.e., chrysin, to the CYP1A2 enzyme was conducted.

**Figure 6:**
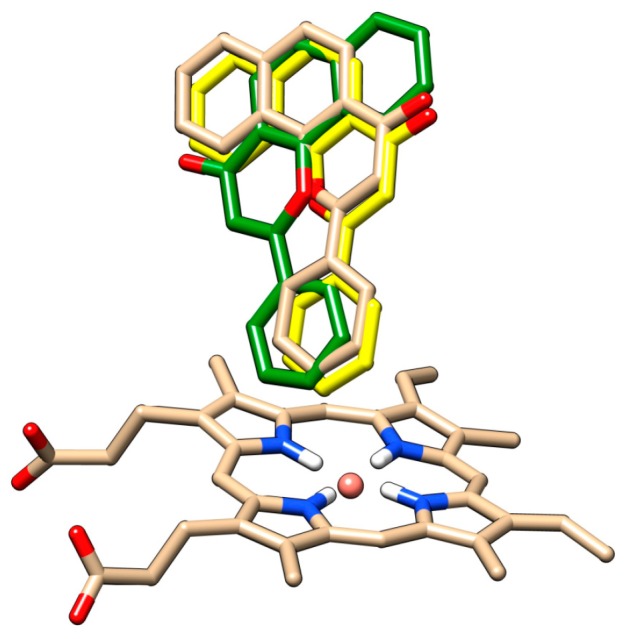
Comparison of the docked alpha-naphthoflavone with all water molecules removed (green), docked alpha-naphthoflavone in presence of HOH 733 (yellow), and crystallographic alpha-naphthoflavone (brown) in vicinity of cytochrome P450 1A2 heme. Oxygen atoms are depicted in red, nitrogen atoms in blue, hydrogen atoms in white, and heme iron in orange.

The redocking of alpha-naphthoflavone, both in presence and in absence of HOH 733, was performed and compared with crystallographic data (Figure 6). The docked positions of alpha-naphthoflavone with and without the water molecule were rotated by approximately 180° compared to each other. Alpha-naphthoflavone was docked with the water molecule having the virtually same coordinates as the crystallographic alpha-naphthoflavone. This was used as a confirmation that the docking of our chrysin species with the HOH 733 water molecule was a valid approach, as the water molecule can affect the ligand position in the active site of CYP1A2 [37,38].

**Figure 7:**
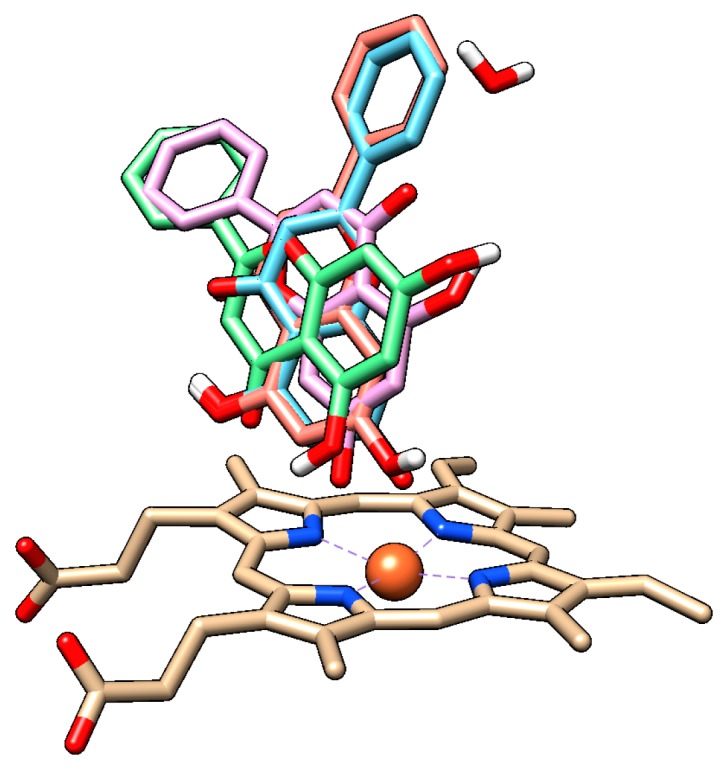
Comparison of docked chrysin species in presence and in absence of the HOH 733 water molecule (the results are identical in both cases). Anion species are depicted in blue (top-ranked docked species) and pink (second top-ranked docked species), while molecule species are depicted in green (top-ranked molecule species) and orange (third top-ranked molecule species in presence of HOH 733 and second top-ranked molecule species in absence of HOH 733). Oxygen atoms are depicted in red, nitrogen atoms in blue, hydrogen atoms in white, and heme iron in orange.

For both chrysin species, docking with and without the HOH 733 water molecule was done and their positions were compared (Figure 7, Table 1).

The anion species at position 7 binds in the same position both in presence and in absence of the HOH 733 water molecule, with the 7-O- group closest to the heme iron. For the docked molecule species, the HOH 733 water molecule does not play a significant role. The top-ranked docking pose in both cases was identical, with the 5-OH and 4-keto group being oriented toward the heme iron. Meanwhile, in the less populated clusters, the group closest to the heme iron was the 7-OH group. The second-ranked cluster of docked molecule species in presence of HOH 733 is not shown, but it was orientated with the B ring facing the heme iron. The binding energy for the molecule species (Table 1) was much lower than for the anion species, suggesting that the molecule species was responsible for most of the chrysin inhibitory effects. Even though the exact values of energy were probably overestimated due to incorrect energy calculations [39], their relative comparison is still possible since we are comparing different poses of the same molecule. The inhibitory constants obtained by computational studies are of similar range to those observed experimentally (micromolar).

In conclusion, out of 11 analyzed flavonoids, hesperetin, pinocembrin, chrysin, isorhamnetin, and morin inhibited CYP1A2 activity; apigenin, tangeretin, galangin, and isorhamnetin inhibited CYP2A6 activity; and chrysin, chrysin-dimethylether, and galangin inhibited CYP2C8. None of the analyzed flavonoids showed inhibition of CYP2D6. The flavonoids in this study were mainly reversible inhibitors of CYP1A2 and CYP2A6, while the inhibition of CYP2C8 was of mixed type (reversible and irreversible). The determined types of inhibition are important for the further assessment of flavonoid-drug interactions. If flavonoid is a reversible inhibitor, flavonoid-rich foods or dietary supplements could inhibit drug metabolism mediated by cytochromes P450 and dose adjustment if needed. If flavonoid is an irreversible inhibitor of cytochrome P450 enzyme, combinations with drugs that it can interact with should be avoided.

## 3. Materials and Methods

### 3.1. Materials

Eleven flavonoids were used in this study. Acacetin, hesperetin, pinocembrin, chrysin, flavone, tangeretin, galangin, isorhamnetin, and morin were acquired commercially from TransMIT (Gießen, Hessen, Germany), while chrysin-dimethylether and tamarixetin were obtained from Extrasynthèse (Genay, Lyon, France). The marker substrates and their corresponding metabolites were purchased from Sigma-Aldrich (St. Louis, MO, USA), except 7-hydroxycoumarin, which was purchased from Extrasynthèse (Genay, Lyon, France).

The recombinant baculosomes with hyperexpressed cytochromes P450 (1A2, 2A6, 2C8, and 2D6) and coexpressed NADPH cytochrome P450 reductase and cytochrome b_5_ were used as a source of enzyme. Baculosomes were commercially obtained from Thermo Fisher Scientific (Waltham, MA, USA), and all other chemicals from Sigma-Aldrich if not otherwise indicated. A 1.0 M solution phosphate buffer of pH 7.4 was prepared in house. NADPH generating system was prepared of 0.1 M glucose-6-phosphate, 10 mg mL^−1^ NADP^+^, and 1000 IU mL^−1^ glucose-6-phosphate dehydrogenase in ratio 100:50:2 (*v*/*v*/*v*) [40].

### 3.2. Determination of CYP1A2 Enzyme Activity

Phenacetine O-deethylation was used as a marker reaction for monitoring CYP1A2 enzyme activity (Figure 2). Incubations of 100 µL were conducted at the temperature of 37 °C. The pH of the incubation mixture was set at 7.4 using a potassium phosphate buffer (final concentration 50 mM). The total amount of CYP1A2 enzyme was 5 pmol, and the final concentration of substrate was 150 µM. Residual activity was determined by incubating the flavonoid (1 µM) with phenacetine as a substrate for 15 min, with or without preincubation, depending of the type of inhibition assay. Reactions were started by the addition of 15 µL of NADPH generating system, while the reactions were stopped by the addition of 1 mL 1% formic acid in acetonitrile. After centrifugation (10 min, 1900× *g*), the clear solution was transferred to a vial for HPLC analysis [22].

HPLC analysis was performed on Agilent 1100 instrument (Santa Clara, CA, USA) coupled with a diode array detector (DAD) on Luna C18 column (4.6 × 150 mm, 3 μm). The gradient method was used for separation. Mobile phase A consisted of water, acetonitrile, and glacial acetic acid in the volume ratio of 90:10:0.1, while mobile phase B contained the same solvents in the ratio 10:90:0.1. The following gradient timetable was used (*t*/min, %B): (0, 0), (2, 0), (13, 55), (14, 0), (20, 0). The flow rate was 1 mL/min, and chromatograms were recorded at 254 nm. Volume of incubation injected to the column was 15 µL. Retention time of substrate was 12.2 min, while product was detected at 5.7 min. The amount of generated product (acetaminophen) was determined as the area under the curve based on the calibration curve of the standard.

### 3.3. Determination of CYP2A6 Enzyme Activity

The aromatic hydroxylation of coumarin at the position 7 was used as a marker reaction for CYP2A6. The incubation conditions were the same as for CYP1A2 enzyme (vide supra) unless otherwise stated. The final concentration of coumarin was 10 µM, while the incubation time was extended to 30 min.

HPLC-DAD analysis was conducted on the same system as the CYP1A2 enzyme with the following modifications: The gradient timetable (t/min, %B) was (0, 20), (1.5, 20), (10, 70), (11, 20), (17, 20), and the detection wavelength was 330 nm. The retention time of the substrate (coumarin) was 7.5 min, while the product (7-hydroxycoumarin) was detected at 4.8 min. The amount of generated product was determined as the area under the curve based on the calibration curve of the standard [22].

### 3.4. Determination of CYP2C8 Enzyme Activity

Paclitaxel 6α-hydroxylation was used as a marker reaction for monitoring CYP2C8 enzyme activity (Figure 2). The incubation conditions were the same as for CYP1A2 enzyme (vide supra) unless otherwose stated. The final concentration of paclitaxel was 20 µM, while the incubation time was set to 30 min.

HPLC-DAD analysis was conducted on the same system as the CYP1A2 enzyme with the following modifications: The gradient timetable (t/min, %B) was (0, 50), (2, 50), (10, 90), (11, 50), (17, 50), and the detection wavelength was 227 nm. The retention time of the substrate (paclitaxel) was 6.1 min, while the product (6α-hydroxypaclitaxel) was detected at 3.4 min. The amount of generated product was determined as the area under the curve based on the calibration curve of the standard [22].

### 3.5. Determination of CYP2D6 Enzyme Activity

O-demethylation of dextromethorphan to dextrorphan was used as a marker reaction for monitoring CYP2D6 enzyme activity (Figure 2). The incubation conditions were the same as the CYP1A2 enzyme (vide supra) unless otherwise stated. The final concentration of the marker substrate in the incubation was 100 µM, while the incubation time was set to 30 min.

HPLC-DAD analysis was conducted on the same system as for CYP1A2 enzyme with the following modifications: The gradient timetable (t/min, %B) was (0, 18), (5, 18), (10, 40), (15, 18), and the detection wavelength was set to 224 nm. The retention time of the substrate (dextromethorphan) was 7.1 min, while the product (dextrorphan) was detected at 4.1 min. The amount of generated product was determined as the area under the curve based on the calibration curve of the standard [22].

### 3.6. Determination of the Inhibition Type

Three types of experiments were conducted to determine metabolism dependent inhibition, time dependent inhibition, and direct inhibition of CYP1A2, CYP2A6, CYP2C8, and CYP2D6 enzymes [40].

To determine metabolism dependent inhibition (MDI assay), the flavonoid was first preincubated with the enzyme with the addition of the generating system for 30 min, after which the marker substrate was added to determine residual enzyme activity (as described above for each enzyme).

If metabolism dependent inhibition was determined, time dependent inhibition and direct inhibition were tested. Time dependent inhibition (TDI assay) was assessed by preincubating the flavonoid and enzyme, without the generating system, after which residual activity was determined by adding the NADPH generating system along with the substrate (as described above for each enzyme).

The direct inhibition assay (DI assay) was conducted without preincubation, i.e., the NADPH generating system was added to the incubation mixture containing flavonoid and substrate following the experimental set-up described above for each individual cytochrome P450 enzyme [40].

If the flavonoid is an irreversible inhibitor of the cytochrome P450, it will decrease enzyme activity in the MDI assay, while no reduction of enzyme activity will be observed in TDI and DI assays. Pure time dependent inhibitors require time to interact with the enzyme. Thus, TDI inhibitors will show a reduction of enzyme activity in assays with preincubations, i.e., the MDI and TDI assays, but not in the DI assay (no preincubation). Pure direct inhibitors are reversible inhibitors that usually compete with the substrate binding to the active site and will show a decrease of enzyme activity with or without preincubation. DI inhibitors will reduce enzyme activity in all three assays (MDI, TDI, and DI).

### 3.7. Docking Studies

To examine the binding of chrysin to the active site of cytochrome P450 1A2, a docking study was performed using AutoDock 4.2.6. (The Scripps Research Institute, La Jolla, CA, USA) [39], which used dispersion, hydrogen bonds, and electrostatic and desolvatation energy components to determine the conformation of the most probable complex. The three-dimensional coordinates of the cytochrome P450 1A2 molecule co-crystallized with alpha-naphthoflavone were obtained from the RCSB [41]. This crystal structure was chosen due to similarity of alpha-naphthoflavone with our ligand, and the crystal structure had a satisfactory resolution of 1.95 Å. The protein molecule was prepared for docking by adding the missing side-chain atoms and hydrogen atoms, all Lys, Arg, His, and Cys side-chains were protonated, all Asp and Glu side-chains were deprotonated, and the amino and carboxy termini were charged. Since there was a presence of a water molecule (HOH 733) in the vicinity of alpha-naphthoflavone molecule in the active site, the docking study was performed both with all water molecules removed, as well as with all water molecules removed except HOH 733. The three-dimensional forms of the ligands were drawn, and their initial geometries were minimized in HyperChem 8.0 (Hypercube, Inc., Gainesville, FL, USA). Their charge was set to represent the most abundant species at pH 7.4, calculated at chemicalize.com. At pH 7.4, chrysin-7-anion represents 65.49% and chrysin molecule represents 11.44% of all chrysin species. Both species were docked due to the fact that the percentage of the molecule species sharply increases with lowering of the pH, as happens in intrahepatic conditions [42]. Partial charges for flavonoid ligands were set according to Ionescu et al. [43]. In AutoDock grid maps of size 70 × 70 × 70 Å were generated with 0.375 Å spacing centered in the CYP1A2 active site cavity (4.0, 12.0, 23.0) by the AutoGrid program [39] and Lamarckian genetic algorithm (LGA) [44] was applied. The receptor molecule was regarded as rigid, while all ligand single bonds were allowed to rotate freely during the Monte Carlo simulated annealing procedure. Ligand flexible docking simulations were performed with 100 runs, population size of 150, 2.5 × 107 energy evaluations, 27,000 numbers of generations, rate of gene mutation of 0.02, and rate of crossover 0.08. A root-mean-square-deviation (RMSD) of 2.0 Å was used as a criterion for cluster analysis of the docking results (in order to determine if two docked conformations were similar enough to be included in the same cluster). First, the docking of alpha-naphthoflavone in presence and in absence of HOH 733 was conducted in order to assess the appropriateness of the system. Afterward, the docking of chrysin species was conducted, also in the presence and absence of HOH 733.

### 3.8. Statistical Analysis

All incubations were conducted in triplicate. The results are expressed as the residual activity of the enzyme, i.e., the percentage of product generated in incubation with the addition of flavonoid in ratio to the control without flavonoid. The statistical significance was tested with Student’s t-test in the program R (The R Project for Statistical Computing, Vienna, Austria).

## Figures and Tables

**Table 1:** Summary of Chrysin Species Docking.

Chrysin Anion at Position 7								
In Presence of HOH 733					In Absence of HOH 733			
Cluster Rank	Percentage of Runs	Binding Energy (kcal/mol)	Inhibitory Constant (μM)		Cluster Rank	Percentage of Runs	Binding Energy (kcal/mol)	Inhibitory Constant (μM)
1.	84	−6.91	8.66		1.	80	−6.94	8.23
2.	16	−6.70	12.22		2.	20	−6.75	11.26
Chrysin Molecule								
In Presence of HOH 733					In Absence of HOH 733			
Cluster Rank	Percentage of Runs	Binding Energy (kcal/mol)	Inhibitory Constant (μM)		Cluster Rank	Percentage of Runs	Binding Energy (kcal/mol)	Inhibitory Constant (μM)
1.	81	−8.23	0.93		1.	98	−8.26	0.89
2.	17	−8.13	1.10		2.	2	−8.09	1.18
3.	2	−8.07	1.21					
